# Chronic Cannabis Intoxication and Propofol-Induced Salivation: Causes and Considerations

**DOI:** 10.3390/pathophysiology29020018

**Published:** 2022-05-28

**Authors:** Allison Derise, Carey Ford, Nazar Hafiz, Sudha Pandit, Aditya Vyas, Samuel Igbinedion, James Morris, Paul Jordan, Qiang Cai, Jonathan Steven Alexander

**Affiliations:** 1 School of Medicine, LSU Health Sciences, Shreveport, LA 71130, USA; ald003@lsuhs.edu (A.D.); clf002@lsuhs.edu (C.F.); 2 Division of Gastroenterology and Hepatology, LSU Health Sciences, Shreveport, LA 71130, USA; nazar.hafiz@lsuhs.edu (N.H.); sudha.pandit@lsuhs.edu (S.P.); aditya.vyas03@lsuhsc.edu (A.V.); samuel.igbinedion@lsuhs.edu (S.I.); james.morris@lsuhs.edu (J.M.); paul.minidoc@gmail.com (P.J.); qiang.cai@lsuhs.edu (Q.C.); 3 Department of Molecular and Cellular Physiology, LSU Health Sciences, Shreveport, LA 71130, USA

**Keywords:** cannabis, propofol, hypersalivation, sedation

## Abstract

Legalization/decriminalization of cannabis will increase the numbers of patients who have had recent exposure to recreational or medical cannabis. Currently, little has been reported about potential interactions between cannabis use and Propofol anesthesia e.g., for oropharyngeal procedures. We describe three cases of ‘cannabis-induced hypersalivation after propofol’ (CHAP) and present our institutions’ experience with this unique pharmacological combination. Increased hypersalivation may complicate procedures and represent a procedural risk of suffocation. We evaluate possible pharmacological interactions that might underlie this phenomenon and consider management options going forward.

## 1. Introduction

Patients with a history of cannabis use often have complications during routine procedures requiring general anesthesia where increased levels of sedatives may need to be administered [1]. At LSUHSC-S, we have observed a paradoxical and dramatic increase in saliva flow in patients intoxicated with cannabis during procedures where Propofol is being administered as a general anesthetic. Our division has recognized a phenomenon known as Cannabis-Propofol salivation (CPS) which appears to reflect pharmacological and physiological effects of chronic cannabis use on anesthesia. This phenomenon appears to occur frequently at our institution and will likely increase in frequency given the more relaxed attitudes towards ‘recreational’ or self-administered cannabis. There is a significant problem in our endoscopy suite with so much saliva being generated during these procedures that patients are in peril of suffocating on these secretions, and greater effort must be maintained by the anesthesia team to guard against ‘drowning’ or inhalation of saliva. While we recognize Cannabis-Propofol hypersalivation as a bonafide phenomenon, the physiology and pharmacology underlying this interaction is unclear and appears to be complex given the well-described and seemingly antagonistic influence of cannabis on saliva output. There are very few if any descriptions of this in the literature that might guide clinical management.

We report three cases of CPS with similar case histories and outcomes. We propose that chronic cannabinoid use leading to higher doses of Propofol during surgical procedures acts synergistically on the salivary glands to provoke excessive intracellular Ca mobilization, thus leading to excessive salivation.

## 2. Case Reports

**Patient 1** was a 58-year-old Caucasian woman seen in 2021 with a past medical history of gastric bypass (Roux En-Y) in 2003 followed by gastrectomy and 10 inches of small bowel resection, osteoarthritis, carpal tunnel syndrome (on Cymbalta and gabapentin), chronic tobacco use (1 pack per day) and Marijuana use. She declared her last use of Cannabis 7 days prior to her GI procedure. She was taking several medications that have no known effects on salivary secretion (Aripiprazole, Atorvastatin, Chlorthalidone, Doxepin, Ergocalciferol, Furosemide, Olmesartan), which produce xerostomia (Estradiol, Omeprazole, Oxybutinin, Vortioxetine). No significant lab values were noted in the Electronic Medical Records (EMR). She presented an esophagogastroduodenoscopy (EGD) for evaluation of gastric ulcers. During the procedure, the patient required a total of 130 mg of Propofol. It was observed the patient was salivating excessively which required frequent suctioning and extra dosing of anesthetic medication. Other than excessive salivation, the patient underwent the EGD without complications and was discharged home after the procedure.

**Patient 2** was a 57-year-old African American man with a past medical history of hypertension (on lisinopril and hydrochlorothiazide), peripheral vascular disease, coronary artery disease s/p Percutaneous Coronary Intervention with stent placement, chronic hepatitis C infection (on epclusa treatment), liver cirrhosis, chronic alcohol use (drinks beer daily) and declares regular marijuana use with his last use 1 day prior to his GI procedure. This patient was taking medications that provoke xerostomia (Amlodipine, Lisinopril, Metoprolol) or which have no known effects on salivary function (Aspirin, Atorvastatin, Ferrous sulfate, Hydrochlorthiazide, Metformin, Spirinolactone). No significant lab values were noted in the Electronic Medical Records (EMR). He presented an esophagogastroduodenoscopy (EGD) for variceal screening. During the procedure the patient required a total of 100 mg of Propofol. It was observed the patient was salivating excessively and required frequent suctioning and extra dosing of anesthetic medication. Other than excessive salivation, the patient underwent the EGD without other complications and was discharged home after the procedure.

**Patient 3** was a 31-year-old Caucasian man with a significant past surgical history of cholecystectomy who presented to the emergency room with complaints of two episodes of blood-tinged vomitus and blood mixed with partially digested food. The patient denied any abdominal pain, dark, tarry stools or alcohol consumption. This patient was using omeprazole and ondansetron, which produce xerostomia or have no known effect on salivation, respectively. No significant lab values were noted in the Electronic Medical Records (EMR). Patient declared frequent marijuana use with a last use 3 weeks prior to GI procedure. After stabilization the patient was discharged and scheduled for an esophagogastroduodenoscopy (EGD) after 2 days as an outpatient. During the procedure the patient required 2 mg of midazolam and 200 mg of Propofol. It was observed the patient was salivating excessively which required frequent suctioning and an extra dose of anesthetic medication. Other than this excessive salivation, the patient underwent the EGD without complication and was discharged home after the procedure.

These patients share the frequent use of cannabis and several other risks, e.g., tobacco and alcohol use, which may predispose them to complications during EGD procedures. However, our division has long been aware of increased risks for hypersalivation during EGD procedures which may be consistent with interactions between Propofol and cannabis. In the following sections we consider the physiology of salivation, the influence of cannabinoids on salivation and the potential interaction between Propofol and cannabis in triggering this response. An increased awareness of this phenomenon and its causes may prevent the development of this clinical response and enable safer patient management during procedures requiring Propofol sedation.

## 3. Physiology of Salivation

The salivary glands produce 1500 mls of saliva daily, which lubricates food, participates in enzymatic digestion and protects the oral corridor against injury and infection [2]. Saliva production by the submandibular gland is greater than the parotid and sublingual glands. Salivary secretion is normally controlled by the autonomic nervous system, mainly the parasympathetic nervous system, which releases acetylcholine in response to central nervous system signals. Parasympathetic nerves responsible for salivation are controlled by the nucleus tractus solitarius. The superior salivary nerve (facial nerve, cranial nerve VII) innervates the submandibular and sublingual glands while the glossopharyngeal nerve (inferior salivary /cranial nerve IX) innervates the parotid glands via the otic and submandibular ganglia [2,3]. Parasympathetic stimulation also increases salivary gland blood flow which increases fluid delivery to these glands [4]. Human labial glands have been reported to express muscarinic M1, M3 and M5 receptors with no or low amounts of muscarinic M2 and M4 receptors detected [5].

**Figure 1:**
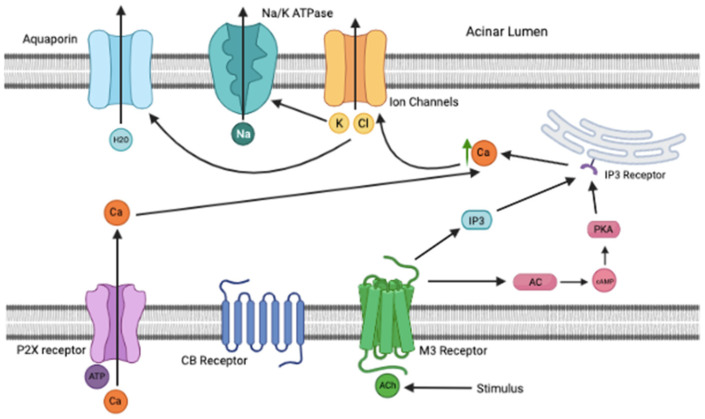
M3 Pathway. A stimulus causes release of Acetylcholine (ACh) which activates M3 receptors. This activation leads to increased levels of IP3 and cAMP, causing intracellular calcium release from the endoplasmic reticulum. Meanwhile, ATP activates the P2X receptor which allows extracellular calcium to move intracellularly and adds to the total intracellular calcium content. The elevated intracellular calcium causes opening of the Chloride ion channel, which then causes sodium and water to follow into the acinar lumen via Na/K ATPase and Aquaporin channels, respectively. Created with BioRender.com (accessed on 1 April 2022).

Cholinergic signaling dramatically affects salivary flow (water, electrolytes) via acinar muscarinic M3 receptors, which has been shown to be the main muscarinic receptors that triggers and regulates saliva secretion and ejection seen in Figure 1 [6,7]. This signaling triggers phospholipase to cleave membrane phospholipids to form inositol triphosphate (IP3) and elevate intracellular calcium via binding of IP3 Ryanodine receptors on the endoplasmic reticulum [8,9]. This increase in calcium opens chloride channels which allow passive chloride flow in the acinar lumen where sodium follows, then water, generating saliva. Cl- transporting proteins expressed at the basolateral aspect of the cells allow Cl- to be released at a higher concentration than its equilibrium potential. The Na/K ATPase pump helps drive Na+ movement to follow chloride and Na/K ATPase inhibition reduces saliva secretion [6]. Water movement via aquaporin-5 protein channels in the salivary glands requires this transcellular secretion of Cl- and this first phase allows acinar cells to generate a plasma-like isotonic salivary fluid. Several channels and transporters ensure unidirectional transport of ions from the serosal (basolateral) aspect to the apical (luminal) side [6,10]. Parasympathetic stimulation triggers the release of a isotonic saliva (relative to interstitial fluid or plasma) from the acini; after passing through the salivary ducts the saliva formed in the acini becomes hypotonic. The isotonic primary saliva fluid is diluted and ionically modified as it passes through the salivary ducts, where most of the sodium and chloride is reabsorbed. In the ducts, Na^+^ is actively reabsorbed while Cl^−^ is passively reabsorbed and K+ and bicarbonate (HCO3^−^) are actively secreted, making the final saliva hypotonic [2,3]. The final K+ concentration in saliva is also higher than in plasma reflecting K+ and HCO3^−^ secretion. The final saliva is typically hypotonic as the ductal epithelium is impermeable to water and Na^+^ and Cl^−^ reabsorption exceeds K^+^ and HCO3^−^ secretion [2,3]. While parasympathetic stimulation is well known to increase saliva formation, parasympathetic denervation can also increase saliva secretion [11], suggesting that this regulation loop is complex.

Sympathetic innervation of salivary glands is considered more minor wherein saliva production is controlled via the superior cervical ganglia. Sympathetic stimulation releases norepinephrine which activates alpha- and beta-adrenergic receptors to decrease saliva production by acinar cells, but increase protein secretion, and decrease blood flow to salivary glands. The protein content (generally enzymes) of saliva is additionally controlled by neuropeptide signaling and by sympathetic nerves via the superior cervical ganglia which deliver norepinephrine to the major salivary glands (parotid, submandibular and sublingual) which increases the viscosity of saliva via increased mucous release [12].

## 4. cAMP in Acinar Cells Synergizes with Calcium to Drive Saliva Secretion

Paradoxically, in acinar cells, cAMP helps to mobilize intracellular calcium, and stimulates secretion salivary flow as well as protein release. Calcium and cAMP have synergistic effects on fluid secretion such that peak secretion is elicited following activation of both parasympathetic and sympathetic pathways [10]. This reflects protein kinase A (PKA)-mediated phosphorylation of inositol 1,4,5-trisphosphate receptors (IP(3)R) in acinar cells which maximally activates calcium release via release from the endoplasmic reticulum [9,10]. Sustained salivary secretion from the acinar cells requires influx of extracellular calcium [7,8], which is where the P2X receptor can come into play. The P2X receptors are a family of ligand-gated ion channels that are stimulated by ATP to allow extracellular calcium into the cell to couple with the calcium released by the endoplasmic reticulum [6]. Reports have noted that ATP stimulation of calcium release was powerfully enhanced via P2X4 activation specifically and triggered inflow of extracellular calcium into the cells [9,10].

## 5. Physiology and Pharmacology of Cannabis on Salivation

Cannabinoid receptors (CB1 and CB2 receptors) are G protein coupled receptors found in mammalian tissues [13]. CB receptors have been described throughout the gastrointestinal tract and are endogenously activated by anandamide (AEA) and 2-arachidonylglycerol (2-AG). These ligands cause reduce gastrointestinal secretion, motility and blood flow [14]. Activation of cannabinoid receptors also enhances central hunger responses (‘munchies’) [15].

**Figure 2:**
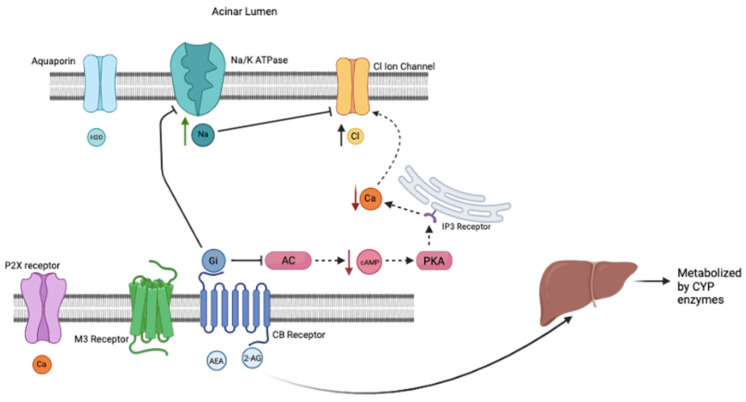
Active Cannabinoid Use Pathway. AEA and 2-AG act on the CB Receptors, thus activating the Gi subunit. This causes downstream inhibitory effects on cAMP, intracellular calcium, the Na/K ATPase pump, and the aquaporin channel. The increased intracellular sodium and decreased intracellular calcium inhibit the chloride ion channel, preventing the release of chloride to form salivation. Because the chloride channel is closed and there is no increased chloride gradient within the lumen, sodium and water do not move into the acinar lumen via their respective channels. Created with BioRender.com (accessed on 1 April 2022).

Cannabinoids are known to reduce salivation (xerostomia/cottonmouth) and CB1 and CB2 receptors have been identified in submandibular glands [16]. Activation of the CB1 receptor in the submandibular gland inhibits sodium transport and subsequently Na/K ATPase transport eliminating the driving force for chloride transport and modulates flow of saliva [17]. CB2 receptors have been reported to influence salivary consistency and composition (Na^+^). Furthermore, cannabinoid receptor activation by AEA was shown to reduce forskolin (adenylate cyclase activator) -induced increase of cAMP in submandibular acinar cells of rats, which would lead to a decrease in salivary secretion [16]. It appears that cannabinoid receptor activation has dual effect on inhibiting both Na/K ATPase and adenylate cyclase, which leads to the intracellular suppression of salivary secretion (see Figure 2), which is normally activated by acetylcholine. Physiologically, AEA also has also been shown to suppress salivation by relaxing the myoepithelial cells surrounding the salivary lumen, and by blocking the release of neurotransmitters in the presynaptic terminals of the submandibular glands [16].

## 6. Habituation/Desensitization of Cannabinoid Receptors

The phenomenon of desensitization may be considered in those who are acute versus chronic cannabinoid users, both of which could have downstream effects on salivation. It is known that repeated intake of cannabis and CB1 agonists can produce tolerance that may be achieved by internalization of CB1 receptors and/or a reduction in the receptor recycling, production or signaling [13]. A study conducted with monoglyceride lipase (MGL) knockout mice, endocannabinoid 2-AG degradation deficient mice, showed no response to CB1 and CB2 receptor agonists consistent with the conclusion that there was a desensitization of CB receptors in knockout mice because these mice had super-elevated levels of endocannabinoid (2-AG) which reduced effects of administered CB receptor agonists. This study confirmed that cannabinoid receptors are internalized in endocytic vesicles and trafficked to lysosomes. An interesting result from this study was an observed difference in desensitization between mice that were acutely versus chronically treated with monoglyceride lipase (MLI) inhibitors. In that study, acute treatment did not desensitize CB1 receptors, an effect that has been observed in mice chronically treated with MLI [18].

Another study also noted that chronic cannabinoid treated animals showed profound desensitization of several signal transduction pathways downstream of cannabinoids. Because net ligand activity reflects numbers of available functional receptors and competent signaling associated with the receptor activation, a reduction in CB1 receptors available for signaling, agonist-induced effects of marijuana would be significantly reduced and may even convert into antagonism [19]. Therefore, chronic cannabinoid use would be anticipated to lead to higher levels of monoglyceride lipase and lower cannabinoid receptor expression. Therefore, individuals chronically using cannabinoids individuals might compensate for xerostomia. In the absence of cannabinoids, habitual cannabis users might be expected to have higher than normal levels of salivation reflecting rebound cAMP and other mechanisms. Because individuals who chronically use cannabis also have higher activity of the CYP450 system to catabolize cannabinoids, this system may influence the rate at which Propofol is metabolized and require higher levels of Propofol to achieve or maintain sedation.

## 7. Effects of Propofol on Salivation

Diprivan (Propofol) is a sedative-hypnotic agent used before and during general anesthesia for medical procedures. Propofol is considered the ‘sine qua non’ sedative for GI endoscopic procedures [20]. Propofol is a positive allosteric modulator of gamma-aminobutyric acid type A receptors and enhances the inhibitory action of gamma-aminobutyric acid (GABA) by increasing the duration of chloride channel opening, leading to hyperpolarization of nerve and other cell membranes [21,22].

Propofol alone or in combination with another drug has been linked to hypersalivation [23]. Propofol has been shown to increase influx of extracellular calcium into the cytoplasm [24,25,26] by potentiating acinar cell responses to ATP mediated by P2X4 receptors expressed on acinar cells. Because of the significant dependence of salivation on calcium, Propofol’s action on cell calcium by these mechanisms may represent a possible mechanism for propofol to increase saliva secretion. The ability of Propofol to increase chloride channel opening may explain some proportion of hypersalivation during propofol sedation. Mean salivation was significantly elevated in patients treated with propofol in combination with remifentanil (an ultra-short-acting synthetic opioid) compared to sevoflurane group. Propofol plus remifenatil has also been shown to decrease plasma chloride, an effect that is consistent with effects of propofol on chloride channels and secretion [23].

## 8. Pharmacokinetics of Cannabis and Propofol

Propofol interactions in individuals using cannabinoids may call for increased dose adjustment for propofol and increased propofol exposure of salivary glands would be expected to occur with greater propofol sedation. Because propofol is a UDP Glucuronosyltransferase (UGT) substrate (UGT1A9), in vitro studies have predicted that inhibition of UGT1A9 by cannabinoids (e.g., cannabidiol) may increase biological persistence potentially resulting in clinically important complications, particularly since a greater loading of propofol may be required to achieve equivalent sedation. Currently, polymorphisms in UGT1A9 and other propofol catabolizing pathways, e.g., CYP2B6 that limit glucuronidation and/or metabolism, may decrease propofol clearance with greater effect [27]. Conversely, chronic exposure to cannabinoids would be expected to increase UTG1A9 expression and increase the rate of propofol clearance by this pathway.

Another pathway driving an increased requirement for anesthetic agents like propofol in cannabis users may be the impact of chronic cannabis exposure on the cytochrome P-450 system. CYP enzymes degrade Cannabinoids and chronic cannabinoid use increases the expression and activity of several CYP components. For example, because cytochrome P-450 2B6 (CYP450-2B6) is a major pathway for hydroxylation/oxidation of propofol [28,29], increased mobilization of CYP450-2B6 following chronic cannabis use may lead to more rapid catabolism of propofol and consequently higher propofol dosing required for equivalent sedation. Elevated levels of propofol following use cannabis necessitated by higher activity UGT and CYP450 pathways may predispose such patients to greater risk for higher propofol levels and perhaps, hypersalivation. This phenomenon has been previously described and showed that cannabis use can necessitate up to twice as much anesthetic (propofol and other anesthetics) to achieve equivalent sedation [1,30]. Such effects that may drive greater saliva production and ejection. In mice, propofol sedation is potently blocked by delta 9-tetrahydrocannabinol (THC) which anticipated higher doses of propofol for equivalent sedation [31]. Animals given both cannabis plus propofol also showed markedly prolonged recovery times after anesthesia (>4-fold longer than with propofol alone) [32].

**Figure 3:**
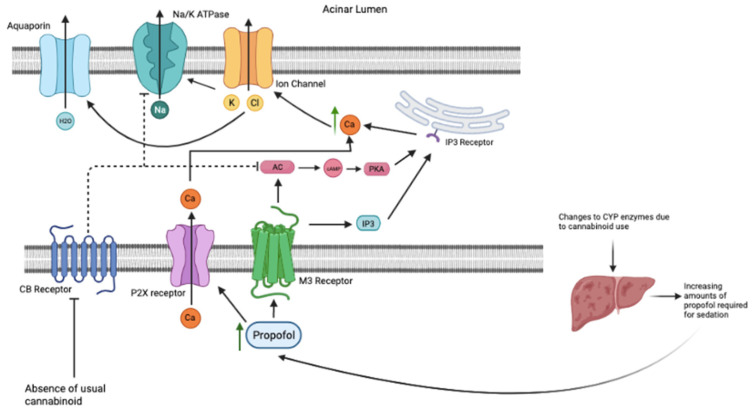
Cannabis Withdrawal and Propofol Administration. The effects of absence of usual cannabis and administration of Propofol cause a synergistic effect. The cannabis withdrawal removes inhibition on the Adenylate Cyclase (AC) pathway and the Na/K ATPase pump. The increased need for administration of Propofol to ensure sedation due to the CYP enzyme changes from the cannabis use causes an increase activation of the AC and IP3 pathways. Propofol also opens the P2X receptor causes extracellular calcium to move into the cell. The increased intracellular calcium opens the chloride ion channel and allows the release of chloride into the acinar lumen. Sodium and water follow the gradient via their respective channels. Created with BioRender.com (accessed on 1 April 2022).

When combined, the influence of Propofol on salivation with the rebound elevations in cAMP and calcium may provoke a scenario as shown in Figure 3, where these effects drive hypersecretion of saliva. This may represent a novel and increasingly more common complication of Propofol sedation. The risk of hypersalivation in a sedated individual can vary from a minor nuisance to a significant and catastrophic event when airway management is compromised.

## 9. Pharmacological Model of Propofol: Cannabis Interactions

The normal salivation pathway involves muscarinic M3 receptor activation, causing adenylate cyclase and IP3 to bind to the IP3 Ryanodine receptor on the endoplasmic reticulum. The endoplasmic reticulum releases calcium into the cell. ATP activates P2X receptor that allows extracellular calcium into the cell. The total increase in intracellular calcium causes the opening of the ion channel, causing chloride and potassium to move into the acinar lumen. This movement activates the Na/K ATPase pump and aquaporin channels to release sodium and water, respectively, into the acinar lumen. When cannabis is used, this pathway is inhibited by the Gi subunit of the CB receptors. This subunit specifically inhibits both adenylate cyclase and the Na/K ATPase pump. This causes calcium to remain in the endoplasmic reticulum and no movement of ions and water into the acinar lumen, causing the famous “cotton mouth” seen with cannabis use. Over time, CYP enzymes within the liver have increased expression and activity, which can affect the metabolism of Propofol. When cannabis is eliminated and Propofol added to the patient, like what would happen on the day of a procedure, the downstream effects are synergistic to saliva production. Propofol activates both the M3 receptor and P2X receptor to activate the cAMP and IP3 pathway to increases intracellular calcium, as well as having extracellular calcium come into the cell. This causes the opening of the ion channel, Na/K ATPase, and aquaporin channels as previously mentioned. Higher doses of Propofol are required to maintain sedation because that cannabis has on the CYP enzymes that are involved in metabolizing Propofol. The withdrawal of cannabis takes away the inhibition on these pathways and channels stated above. Both occurring together may cause hypersalivation and pose risks to patients undergoing such procedures. To help combat this risk, atropine and glycopyrrolate could be administered IM or IV in premedication to minimize salivation. While the use of atropine or glycopyrrolate to suppress profuse salivation has been proposed, it risks complications with polypharmacy involving cannabis, Propofol, atropine and glycopyrrolate.

## 10. Discussion

A huge barrier to research and evidence-based knowledge on cannabis has been the long-term illegal status of marijuana which is now followed by its widespread legalization without a full appreciation of the many effects of cannabinoids on drug interactions, conditions, and medical procedures. At our institution, we have anecdotally noticed a correlation in patients with a history of cannabis use and hypersalivation during GI endoscopic procedures. Although we have presented a simple set of pharmacological pathways which may explain this epigenetic phenomenon, the tendency to develop ‘cannabis-induced hypersalivation after Propofol’ (CHAP) may additionally reflect individual metabolic genotype/phenotype, the frequency and type of cannabis product used and the time since the last use of cannabis.

Appreciation of these pharmacological mechanisms might help to explain and prevent this clinical phenomenon in cannabis users that will increasingly be seen during sedation with Propofol. The anticipated management of this unique hypersalivation phenomenon using anticholinergics or cannabis antagonists may provoke more complex problems associated with polypharmacy and may not be recommended because of risks from cardiopulmonary concerns. The identification of cannabis use in patient undergoing Propofol sedation could help to limit the amount of Propofol administered and prevent CHAP.

## Data Availability

Not applicable.
